# Ebstein’s Anomaly: How to Identify Intracardiac Structures When Electrical Signals Do Not Match the Anatomy

**DOI:** 10.19102/icrm.2022.130906

**Published:** 2022-09-15

**Authors:** Lisa Albertini, Andrew C.T. HA, Krishnakumar Nair

**Affiliations:** ^1^Division of Cardiology, University Health Network, Toronto General Hospital, Toronto, Ontario, Canada

**Keywords:** Accessory pathway, CartoSound, congenital heart disease, Ebstein’s anomaly, intracardiac echocardiography

## Abstract

In patients with Ebstein’s anomaly, the distorted anatomy with discordance between the true atrioventricular (AV) groove and the tricuspid valve poses many challenges to the electrophysiologist. Intracardiac echocardiography is a recent tool that allows visualization of the displaced tricuspid valve, the true AV groove, and the atrialized right ventricle. We present a 3-dimensional electroanatomic map built using intracardiac echocardiography and the CARTOSOUND^®^ module (Biosense Webster, Diamond Bar, CA, USA) in one such patient who underwent ablation of a right-sided mid-septal accessory pathway.

Ebstein’s anomaly (EA) is a genetic condition characterized by apical displacement of the septal and posterior leaflets of the tricuspid valve.^1^ In this condition, the distorted anatomy with discordance between the true atrioventricular (AV) groove and the tricuspid valve poses many challenges to the electrophysiologist. Accessory pathways (APs) insert in the true AV groove, but difficult electrogram (EGM) analysis with low-amplitude fractioned signals recorded in the atrialized right ventricle (RV) and a prominent ridge in the groove^2^ can create many challenges in mapping and ablation. The AV groove has been defined in the past with a microelectrode catheter in the right coronary artery and right atrial (RA) angiography.^3^ We used intracardiac echocardiography (ICE) and a dedicated module (CARTOSOUND^®^; Biosense Webster, Diamond Bar, CA, USA) to delineate the right AV anatomy. In **Figure 1**, we present a 3-dimensional electroanatomic map of a 50-year-old patient with EA who underwent a successful right-sided mid-septal AP ablation. The locations of the true annulus where AV signals were recorded (white dots) and a mid-septal AP that was ablated (the red dots indicate the lesion set, and the blue dot indicates the successful ablation site) are shown; the yellow dots represent the His-bundle potential. The green lines display the contour of the RA marked on an ICE image **(Figure 1C)**, and the purple dots indicate the hinge points of the tricuspid valve leaflets. The RA profile is consistent with the RA opacification on the angiogram image. A significantly enlarged RA is seen. **Figure 1D** includes the baseline 12-lead electrocardiogram with ventricular pre-excitation, particularly evident in leads I and III, which disappears after ablation; intracardiac signals at the successful ablation site are also displayed. Use of ICE should become a routine practice in patients with abnormal hearts like those with EA, in whom EGMs do not match the anatomy, to help the electrophysiologist in the challenging task of navigating catheters.

## Figures and Tables

**Figure 1:**
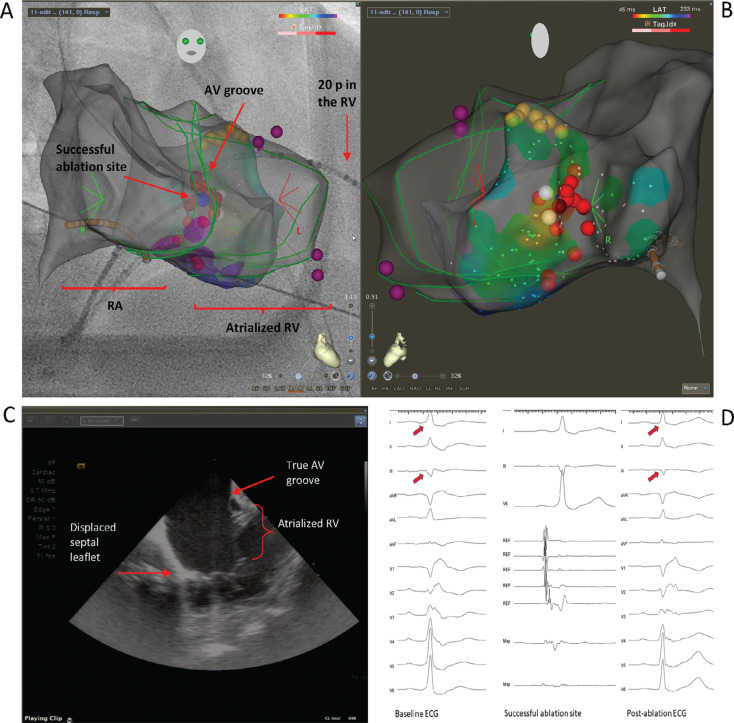
**A and B:** A 3-dimensional electroanatomic map of the right atrium (RA). **A:** Right anterior oblique view. **B:** Left anterior oblique view. The white dots represent the true atrioventricular (AV) groove based on AV signals and the red dots indicate the lesion set, while the blue dot shows the successful ablation site; the yellow dots mark the His-bundle potential. The green lines indicate the contour of the RA marked on ICE, and the purple dots indicate the hinge points of the tricuspid valve leaflets. **C:** Intracardiac echocardiography image of the RA and right AV annulus. **D:** Baseline 12-lead electrocardiogram with ventricular pre-excitation evident in leads I and III **(left)**, intracardiac signals at the successful ablation site **(middle)**, and post-ablation electrocardiogram showing no pre-excitation **(right)**. *Abbreviations:* AV, atrioventricular; ECG, electrocardiogram; Map, ablation catheter; RA, right atrium; REF, coronary sinus catheter; RV, right ventricle.
